# Cancer Cell Response to Anthracyclines Effects: Mysteries of the Hidden Proteins Associated with These Drugs

**DOI:** 10.3390/ijms131215536

**Published:** 2012-11-22

**Authors:** Jirina Tyleckova, Rita Hrabakova, Katerina Mairychova, Petr Halada, Lenka Radova, Petr Dzubak, Marian Hajduch, Suresh J. Gadher, Hana Kovarova

**Affiliations:** 1 Institute of Animal Physiology and Genetics AS CR, v.v.i., 277 21 Libechov, Czech Republic; E-Mails: tyleckova@iapg.cas.cz (J.T.); hrabakova@iapg.cas.cz (R.H.); mairychova@iapg.cas.cz (K.M.); 2 Institute of Microbiology AS CR, v.v.i., 142 20 Prague, Czech Republic; E-Mail: halada@biomed.cas.cz; 3 Laboratory of Experimental Medicine, Institute of Translational and Molecular Medicine, Faculty of Medicine and Dentistry, Palacky University and University Hospital, 775 15 Olomouc, Czech Republic; E-Mails: avodar@gmail.com (L.R.); dzubakp@gmail.com (P.D.); marian.hajduch@upol.cz (M.H.); 4 Life Technologies, Frederick, MD 21704, USA; E-Mail:gadhersuresh@hotmail.com

**Keywords:** anthracycline/anthracenedione, T-lymphoblastic leukemia, proteomics, early anti-cancer response, adaptive cancer mechanisms, protein biosynthesis, ubiquitin-proteasome system, energy metabolism, transport proteins, tumor immunity

## Abstract

A comprehensive proteome map of T-lymphoblastic leukemia cells and its alterations after daunorubicin, doxorubicin and mitoxantrone treatments was monitored and evaluated either by paired comparison with relevant untreated control and using multivariate classification of treated and untreated samples. With the main focus on early time intervals when the influence of apoptosis is minimized, we found significantly different levels of proteins, which corresponded to 1%–2% of the total amount of protein spots detected. According to Gene Ontology classification of biological processes, the highest representation of identified proteins for all three drugs belong to metabolic processes of proteins and nucleic acids and cellular processes, mainly cytoskeleton organisation and ubiquitin-proteasome pathway. Importantly, we observed significant proportion of changes in proteins involved in the generation of precursor metabolites and energy typical for daunorubicin, transport proteins participating in response to doxorubicin and a group of proteins of immune system characterising response to mitoxantrone. Both a paired comparison and the multivariate evaluation of quantitative data revealed daunorubicin as a distinct member of the group of anthracycline/anthracenedione drugs. A combination of identified drug specific protein changes, which may help to explain anti-cancer activity, together with the benefit of blocking activation of adaptive cancer pathways, presents important approaches to improving treatment outcomes in cancer.

## 1. Introduction

**Figure 1:**
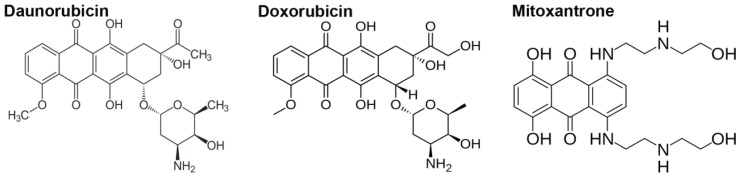
Chemical structures of daunorubicin, doxorubicin and mitoxantrone.

The anthracycline antibiotics doxorubicin (DOXO) and daunorubicin (DNR) belong to the most effective anti-cancer drugs. They have been widely used in clinics for the treatment of both solid tumors and hematological malignancies since the early 1960s, when these products of *Streptomyces peucetius* were first isolated [1]. Structurally, there is only a subtle difference between DNR and DOXO in the side chain of the molecules [2] and mitoxantrone (MTX), an anthracenedione, has also very similar structure to that of anthracyclines [3] (Figure 1).

The mechanism of action of these drugs is attributed mainly to the inhibition of topoisomerase II activity. Topoisomerase II binds to DNA and allows its cleavage but this covalent complex is trapped in the presence of anthracycline drug and DNA cannot re-ligate, thus subsequently blocking transcription and replication [4,5]. Other proposed mechanisms of action are DNA intercalation and the production of reactive oxygen species [6], which appears to be responsible for the serious toxic side effects of these chemotherapeutic drugs, namely cardiotoxicity [7,8]. Despite the similarity in the structure of anthracyclines and anthracenediones, they differ widely in clinical use. DOXO has the widest spectrum of activity amongst anthracyclines and is used for the treatment of both solid tumors and hematological malignancies. It is administered as a single agent or in combination chemotherapy regimens. On the contrary, DNR shows activity mainly in acute leukemia’s [9]. MTX is active both in solid tumors and leukemia with slightly lower activity than DOXO but also with lower toxicity [3]. Even though these drugs are frequently used in clinics, the exact molecular mechanisms of their effects on tumor cells, as well as toxicity, are not completely understood. Importantly, such deeper knowledge might contribute to the clarification of different therapeutic efficiency of structurally very close groups of anthracyclines and anthracenediones.

Proteomic approaches involving gel-based techniques, gel-free chromatography and advanced mass spectrometry for protein fractionation, identification and quantification, allow us to study the effects of drug treatments on cells at protein level in a comprehensive way. The main advantage of 2-D gel based fractionation is the high resolution including assessment of multiple forms of individual protein (s) on the basis of differences in isoelectric point and molecular mass. Using a suitable protein stain, this popular and reliable technique may facilitate comprehensive quantification [10]. Several proteomic studies have been recently performed for monitoring the effect of DOXO on hepatocellular carcinoma [11], breast cancer [12], non-Hodgkin lymphoma [13], acute lymphoblastic leukemia cells [14] or the effect of DNR on pancreatic carcinoma [15]*in vitro.* In addition, proteomic techniques have been used for studying drug resistance mechanisms to DOXO or MTX in lung cancer cells [16,17].

In this study, we performed proteomic comparison of very early effects of DNR, DOXO and MTX treatments on T-lymphoblastic leukemia cells as representative of hematological malignancies. The main goals have been to characterise and identify typical markers of cell response to individual drugs, to define biological processes responsible for their anti-tumor activity and to compare the effects of these structurally linked drugs in order to explain their different therapeutic effectiveness in clinics.

## 2. Results

### 2.1. Determination of IC_50_, TA_50_

Our intention was to investigate the early effects of the anthracycline/anthracenedione anti-cancer drugs that precede the onset of apoptosis in CEM cells and loss of cell viability. The IC_50_ of drugs were determined using the MTT test as mentioned above. The induction of apoptosis in cells began at different time intervals for different drugs. It was therefore necessary to measure time to onset of apoptosis (TA) at first and then to adjust the time of the treatments for each individual drug to the half time of TA (TA_50_). Hence, for all proteomic experiments the cells were treated with 10× IC_50_ doses of the drugs for time interval corresponding to TA_50_ (Table 1). This combination of dose and time of the treatment led to measurable changes in protein composition before onset of apoptosis in treated cells.

### 2.2. Proteome 2-D Maps—Number of Spots per Gel and Number of Differentially Abundant Spots per Anti-Cancer Drug

**Figure 2:**
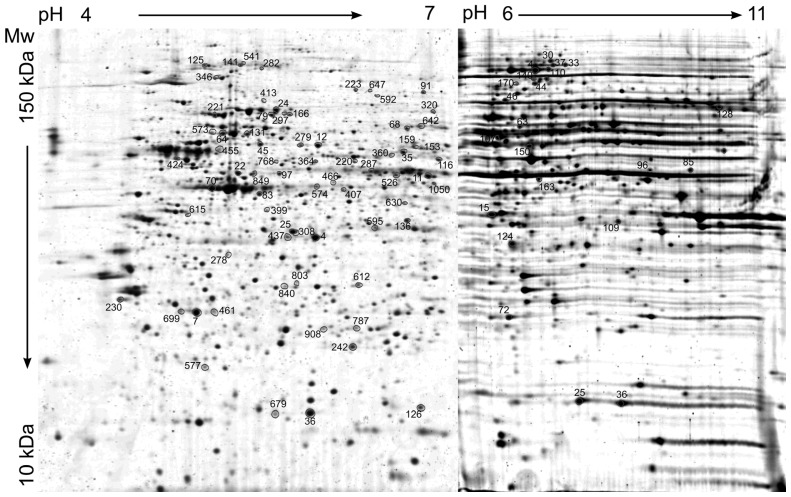
Representative 2-D protein map of treated CEM T-lymphoblastic leukemia cells.

In order to cover the most significant part of the cancer cell proteome, two different pH ranges, pH 4–7 and pH 6–11, were used. The 2D gel images were analyzed using Redfin Solo SW protocol. In this approach, spot detection and image segmentation takes place in a composite image and the same spot positions and borders are then assigned to all images, after compensation for geometric distortions. On average, 2180 and 570 protein spots were detected in pH 4–7 and pH 6–11, respectively (Figure 2). In total for all five anticancer drugs in this study, 133 protein spots showed significantly increased intensity pattern after drug treatment, while 86 protein spots were decreased according to criteria of fold-change >1.2 for *p*-value <0.01 and fold-change >1.5 for *p*-value <0.05. Amongst these, 47 protein spots occurred at different levels in DOXO treatment, 40 protein spots in DNR treatment and 54 protein spots in MTX treatment. Differentially expressed protein spots were selected for mass spectrometry identification and 153 proteins were identified in 174 protein spots which were excised out of all 219 significantly different spots (Table 2). Amongst the identified proteins, there were seven proteins present in two spots and six proteins present in three spots. Contrary to this, two proteins in one spot were identified for seven spots (Table S1). More detailed data regarding mass spectrometry protein identifications including spot number, protein name, UniProt database number, number of peptides matched to the identified protein, number of unassigned peaks, sequence coverage, Mascot score of the identified protein, Mascot score for the highest ranked hit to a non-homologous protein, peptide sequences confirmed by MS/MS (Mascot score given in parenthesis), MW and pI are reported in Table S1.

On average, 2180 protein spots could be detected on pH 4–7 gels and 570 protein spots could be detected on pH 6–11 gels. The spot numbers indicate significantly altered protein spots after daunorubicin, doxorubicin or mitoxantrone treatments (fold change >1.2 and *p*-value <0.01 and fold change >1.5 and *p*-value <0.05). Gels were stained using Sypro Ruby and Redfin SW was used for 2-D gel image analysis.

### 2.3. The Proteins Significantly Changing Their Abundance after Treatment by Individual Anthracycline/Anthracenedione Drugs and Their Distribution by Biological Processes

**Figure 3:**
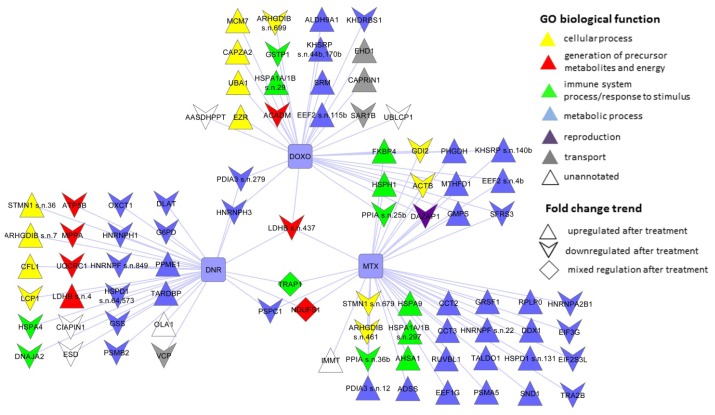
Graphical representation of identified differentially abundant proteins after doxorubicin (DOXO), daunorubicin (DNR) and mitoxantrone (MTX) treatment of CEM T-lymphoblastic leukemia cells.

The proteins significantly changing their abundance and identified as single protein per protein spot for DNR, DOXO and MTX treatments and their classification into biological processes are in Table 3 and depicted in Figure 3. With regard to relatively short time intervals of individual drug treatments, observed increase or decrease in protein levels may be due to impact of drug on turn-over of these proteins.

Light blue squares represent anti-cancer drugs. The nodes show identified proteins marked according to their gene names, the color code represents Gene Ontology biological process based on PANTHER classification. The node shape shows trend of change in protein level, proteins with increased levels are depicted as triangles, proteins with decreased levels as arrowheads and proteins with opposite changes between different drugs as diamonds. Detailed information about the proteins is shown in Table 3.

#### 2.3.1. DNR Induced Protein Changes

**Figure 4:**
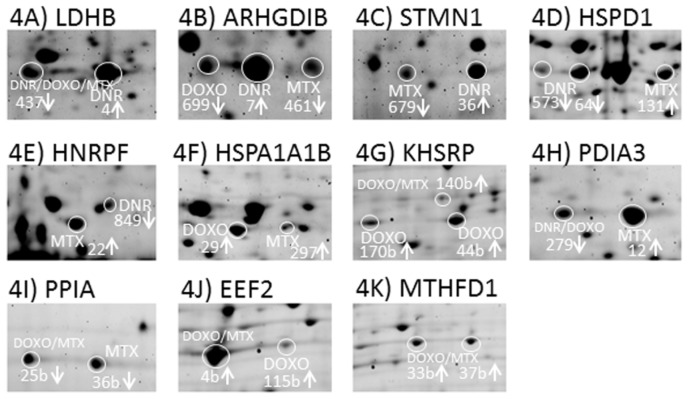
Distinct protein variants of several individual proteins after treatment of CEM T-lymphoblastic leukemia cells with doxorubicin (DOXO), daunorubicin (DNR) and mitoxantrone (MTX).

**Figure 5:**
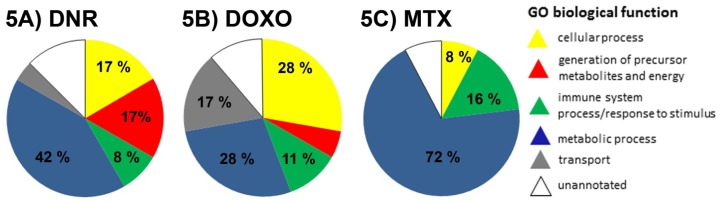
Distribution of anthracycline/anthracenedione regulated proteins by biological processes.

Based on the evaluation criteria applied in this study we have identified 24 proteins at different levels after DNR treatment in CEM cells (Table 3A, Figure 3). Among them, five proteins (l-lactate dehydrogenase B chain, LDHB, spot no. 4; Rho GDP-dissociation inhibitor 2, ARHGDIB, spot No. 7; stathmin, STMN1, spot No. 36; 60 kDa heat shock protein, HSPD1, spots No. 64 and 573; heterogeneous nuclear ribonucleoprotein F, HNRNPF, spot No. 849) represented protein variants specifically affected by DNR whilst another protein forms of these individual proteins observed as distinct protein spots on 2DE were also regulated by DOXO or MTX (Figure 4A–E). Only for HSPD1 there were two protein forms separated by 2DE significantly changed after DNR treatment (Figure 4D). The annotations of the identified proteins in terms of their integration into biological processes according to Gene Ontology implemented in PANTHER software tool were used to classify DNR associated changes in treated cells. The proteins involved in metabolic processes represented 42% of total changes followed by 17% of proteins participating in cellular processes as well as 17% of proteins regulating generation of precursor metabolites and energy (Figure 5A). Interestingly, majority of proteins of metabolic processes were seen to decrease after DNR treatment which is opposite to what we observed for DOXO and MTX (Figure 3). The most expressed DNR induced changes in metabolic processes include decreased levels of glucose-6 phosphate 1-dehydrogenase (G6PD, spot no. 107 b), dihydrolipoyllysine-residue acetyltransferase component of pyruvate dehydrogenase complex (DLAT, spot No. 166), the important part of glycolysis, and glutathione synthetase (GSS, spot No. 768). Additionally, decrease of two heterogeneous nuclear ribonucleoproteins (HNRNPH1, spot No. 220 and a variant of HNRNPF, spot No. 849) involved in mRNA processing was observed. There were only two proteins belonging to the group of metabolic processes with increased levels after DNR treatment, protein phosphatase metylesterase 1 (PPME1, spot No. 407) and TAR DNA-binding protein 43 (TARDBP, spot No. 574). Cellular processes involved in DNR effect were represented by one decreased level of protein, plastin-2 (LCP1, spot No. 221), and three increased levels of proteins including cofilin-1 (CFL1, spot No. 126), STMN1 and ARHGDIB. Common targets of these proteins are actin cytoskeleton and microtubule filaments and their organization. The proteins of group of generation of precursor metabolites and energy appeared to be typical for DNR (Figure 5A) with their only negligible proportion observed after MTX and DOXO treatments (Figure 5B,C). This group consisted of three decreased mitochondrial proteins such as ATP synthase subunit beta (ATP5B, spot No. 424), mitochondrial-processing peptidase subunit alpha (PMPCA, spot No. 360) and cytochrome b-c1 complex subunit 1 (UQCRC1, spot No. 97) as well as increased isoform of LDHB (spot No. 4).

Protein variants were represented by different protein spots of the same protein and are marked with 2DE spot numbers. Arrows indicated trend of protein level changes after drug treatment. 4(A): l-lactate dehydrogenase B chain, LDHB, spot no. 4 was increased by DNR treatment and spot no. 437 was decreased by all three DNR, DOXO and MTX treatments; 4(B): Rho GDP-dissociation inhibitor 2, ARHGDIB, spot No. 7 was increased by DNR, spot No. 699 was decreased by DOXO and spot No. 461 was decreased by MTX; 4(C): stathmin, STMN1, spot No. 36 was increased by DNR and spot No. 679 was decreased by MTX; 4(D): 60 kDa heat shock protein, HSPD1, spots No. 64 and 573 were decreased by DNR and spot No. 131 was increased by MTX; 4(E): heterogeneous nuclear ribonucleoprotein F, HNRNPF, spot No. 849 was decreased by DNR and spot No. 22 was increased by MTX; 4(F): heat shock 70 kDa protein 1A/1B, HSPA1A1B, spot No. 29 was increased by DOXO and spot No. 297 was increased by MTX; 4(G): Far upstream element-binding protein 2, KHSRP spots No. 44b and 170b were increased by DOXO and spot No. 140b was increased by both DOXO and MTX treatment; 4(H): protein disulfide isomerase A3, PDIA3, spot No. 12 was increased by MTX and spot No. 279 was decreased by DNR and DOXO treatment; 4(I): peptidyl-prolyl cis-*trans* isomerase A, PPIA, spot No. 36b was decreased by MTX and spot No. 25b was decreased by both DOXO and MTX; 4(J): elongation factor 2, EEF2, spot No. 4b was increased by MTX and DOXO and spot No. 115b was increased solely by DOXO treatment; 4(K): *C*-1-tetrafydrofolate synthase, MTHFD1, spots No. 33b and 37b were increased by DOXO and MTX treatments.

Pie charts of Gene Ontology classification of biological processes based on the contribution of proteins differentially abundant after treatment of CEM cells by: 5(A) daunorubicin (DNR); 5(B) doxorubicin (DOXO); 5(C) mitoxantrone (MTX).

#### 2.3.2. DOXO Induced Protein Changes

In total, we found 18 proteins significantly changed after treatment of CEM cells by DOXO (Table 3B, Figure 3). Four of these proteins (Heat shock 70 kDa protein 1A/1B, HSPA1A1B, spot No. 29; Far upstream element-binding protein 2, KHSRP spots No. 44b and 170b; ARHGDIB, spot No.699 and elongation factor 2, EEF2 spot No. 115 b) were identified from the protein spots specifically influenced by DOXO although another variants of these proteins were also identified from distinct protein spots which were regulated by DNR or MTX treatment (Figure 4B,F,G,J). KHSRP was found in two evidently separated 2DE spots thus representing multiple forms of this protein (Figure 4G). As regards Gene Ontology classification of identified proteins and their incorporation into biological processes, the proteins involved in metabolic processes represented 28% of total changes and the same percentage was observed for cellular processes, followed by 17% of transport proteins and 11% of proteins from the group of immune system process and response to stimuli (Figure 5B). Metabolic processes were represented by decrease in KH domain-containing, RNA binding, signal transduction-associated protein 1 (KHDRBS1, spot No. 61b) which is an important adapter protein in signal transduction as well as regulator of RNA stability. Furthermore, we found three proteins with increased levels after DOXO treatment including KHSRP, spermidine synthase (SRM, spot No. 278), and EEF2. Among the proteins of cellular processes, there was significant decrease in ARHGDIB and increased expression of three proteins, namely ezrin (EZR, spot No. 592, ubiquitin-like modifier-activating enzyme 1 (UBA1, spot No. 282), and DNA replication licensing factor MCM7 (MCM7, spot No. 91). Transport proteins were observed as selective group of proteins responding to DOXO treatment. They were represented by lowered GTP-binding protein SAR1b (SAR1B, spot No. 908), and higher levels of EH domain-containing protein 1 (EHD1, spot No. 63b) and caprin 1 (CAPRIN1, spot No.141), stress granule associated protein.

#### 2.3.3. MTX Induced Protein Changes

We have identified 25 proteins differentially abundant in CEM T-lymphoblastic leukemia cells followed by MTX treatment (Table 3, Figure 3). Among them there were seven proteins (protein disulfide isomerase A3, PDIA3, spot No. 12; HNRNPF, spot No. 22; peptidyl-prolyl cis-*trans* isomerase A, PPIA, spot No. 36b; HSPD1, spot No. 131; HSPA1A1B, spot No. 297; ARHGDIB, spot No. 461; *STMN1*, spot No. 679) presented as MTX specific protein variants despite distinct forms recognized after DNR or DOXO treatment (Figure 4B–F,H,I). For MTX treatment the proportion of the proteins involved in metabolic processes was the highest observed among DNR, DOXO and MTX drugs and covered 72% of total changes followed by 16% of proteins of immune system process and response to stimuli. Only 8% of proteins involved in cellular processes represented the lowest contribution of this category among DNR, DOXO and MTX drugs (Figure 5C). Amongst the proteins of metabolic processes only a few decreased proteins were observed including transformer-2 protein homolog beta (TRA2B, spot No. 615) and heterogeneous nuclear ribonucleoprotein A2/B1 (HNRNPA2B1, spot No. 109b) driving mRNA splicing and mRNA processing, as well as eukaryotic translation initiation factor 2 subunit 3 (EIF2S3L, spot No. 128b) (Figure 3). The changes of the majority of increased proteins from the metabolic group were mostly moderate with a fold change around 1.4. The most pronounced change observed was the increase in proteosome subunit alpha type (PSMA5, spot No. 230) with a fold change of 2.51, and RuvB-like 1 protein (RUVBL1, spot No. 116) as well as G-rich sequence factor (GRSF1, spot No. 455) (Table 3). Beside metabolic proteins, emerging MTX selective group of proteins of immune system process and response to stimuli was evident (Figure 5C). Majority of these proteins were increased including activator of 90 kDa heat shock protein ATPase homolog 1 (AHSA1, spot no. 83), stress-70 protein (HSPA9, spot No. 24), and HSPA1A1B. Small proportions of proteins of cellular processes were characterized by evident decrease of STMN1 and also lower level of ARHGDIB (Figure 3).

### 2.4. The Protein Changes Linking the Effects of Anthracycline/Anthracenedione Drugs DNR, DOXO, and MTX

In order to evaluate similarities among studied anthracycline/anthracenedione anti-cancer drugs, we looked for the overlap of the proteins changed after treatments. The highest number of such shared proteins was revealed for DOXO and MTX (Table 3, Figure 3). Three proteins, including EEF2 (spot No. 4b), PPIA (spot No. 25b) and KHSRP (spot No. 140b) were also present in another distinct spots affected exclusively either by DOXO or MTX treatment (Figure 4G,I,J. The enzyme *C*-1-tetrahydrofolate synthase (MTHFD1, spots No. 33b and 37b) was present in two spots for both DOXO as well as MTX treatments (Figure 4K). Among these twelve proteins common for DOXO and MTX (Figure 4), the fold changes of increased proteins from the category of metabolic processes ranged between 1.47 and 1.9 including EEF2, MTHFD1, GMP synthase (GMPS, spot No. 46b), d-3-phosphoglycerate dehydrogenase (PHGDH, spot No.153) and KHSRP. Only one protein from this category, splicing factor, arginine/serine-rich3 (SFRS3, spot No. 242) was decreased (Table 3). The group of proteins of immune system process and response to stimuli consisted of two functionally different proteins with isomerase activity PPIA and peptidyl-prolyl cis-*trans* isomerase FKBP4 (FKBP4, spot No. 45) with opposite direction of protein change. Additionally, increased heat shock protein 105 kDa (HSPH1, spot No. 541) was seen to be a part of this group too (Figure 3). Small proportion of cellular processes was directed to regulation of cytoskeleton organization mediated by decreased actin (ACTB, spot No. 70) and small GTP signaling protein Rab GDP dissociation inhibitor beta (GDI2, spot No. 526). Interestingly, a decrease in one protein was observed in this study which was DAZ- associated protein 1 (DAZAP1, spot No. 85b) which belongs to the category of reproduction (Figure 3).

Compared to the overlapping of twelve different proteins for DOXO and MTX treatments, the numbers of common protein overlaps for DNR/MTX and DNR/DOXO were three and two, respectively, with only one protein common for the effect of all three drugs (Table 3 and Figure 3). The proteins common for DNR and MTX included paraspeckle component 1 (PSPC1, spot No. 642), which decreased with evidently high values of fold change for both drugs. On the contrary, two other proteins, heat shock protein 75 kDa (TRAP1, spot No. 320) and NADH-ubiquinone oxidoreductase 75 kDa subunit (NDUFS1, spot No. 413) exhibited opposite trend in protein level showing a decrease after DNR treatment and an increase induced by MTX (Table 3, Figure 3). Different abundance of two proteins of metabolic processes shared between DNR and DOXO regarded heterogeneous nuclear ribonucleoprotein H3 (HNRNPH3, spot. No. 15b) and protein disulfide-isomerase A3 (PDIA3, spot No. 279) (Table 3, Figure 3). The enzyme from the group of generation of metabolic precursors and energy, LDHB (spot No. 437) was significantly decreased after treatment with anthracyclines DNR and DOXO as well as anthracenedione MTX. Interestingly as mentioned above, this enzyme was also identified from protein spot No. 4 increased in response to DNR (Table 3, Figure 3, Figure 4A).

### 2.5. The Proteins Commonly Affected by Five Anti-Cancer Drugs: Anthracycline/Anthracenedione DNR, DOXO, MTX and Distinct Chemotherapeutics CisPt and TAX

Comparison of all five anti-cancer treatments is depicted in Figure S1. The response to cisplatin (CisPt) is presented by 19 unique proteins (Table S2) whilst effect of paclitaxel (TAX) is characterized by 13 proteins (Table S2). Nevertheless, the main purpose of this part of our study was selection of proteins overlapping between CisPt, TAX and anthracycline/anthracenedione drugs (Table S2) to underline common protein features of anti-cancer response. Four proteins (EHD1, spot no. 63b; Medium-chain specific acyl-CoA dehydrogenase, mitochondrial, ACADM, spot No. 163b; KHSRP, spot No. 170b; EZR, spot. No. 592) overlapped for CisPt and DOXO treatments and two proteins (4-trimethylaminobutyraldehyde dehydrogenase, ALDH9A1, spot No. 364; ARHGDIB, spot. No. 699) were shared between TAX and DOXO treatments. Another eight proteins (PPIA, spot No. 25b; CFL1, spot No. 126;, HNRNPA2B1, spot No. 109b; NDUFS1, spot No. 413; PHGDH, spot No. 153, G6PD, spot No. 107b; PPME1, spot No. 407; and TRA2B, spot No. 615) were common for one of the anthracycline/anthracenedione drugs and CisPt or TAX.

### 2.6. Principal Component Analysis of Quantitative Data

**Figure 6:**
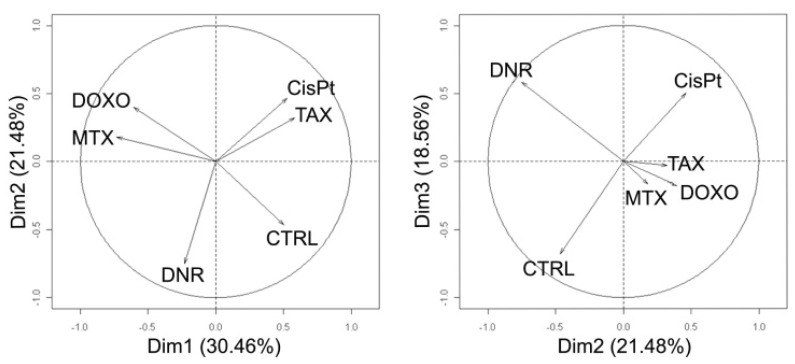
Multivariate principal component analysis of quantitative 2DE data to classify anti-cancer treatments.

Besides pair comparison of protein alterations induced by each treatment, unsupervised multivariate classification (PCA) was performed to provide an overview of the variance in the whole data set including all studied drugs. PCA reduces the huge amount of data into several components named principal components (PCs) on the basis of similarities in the data set. When visualized in two dimensional graphs, the objects/samples with similar behavior tend to “sit together” whilst distance in the position indicates dissimilarity. The first PC accounted for approximately 30% of the total variance in the data, whilst the second PC accounted for approximately 21% of total variance and finally the third PC for nearly 19% of variance (Figure 6). In the first dimension, DNR, DOXO and MTX were separated from CTRL, CisPt and TAX. In the second dimension, DNR separated mainly from DOXO and MTX. Finally, in the third dimension, DNR was more sequestered from CTRL untreated cells.

Principal component analysis was performed to obtain an overview of the variance in the data set and classify treatments according to their similarities or dissimilarities. Quantitative 2DE data of control untreated cells (CTRL), anthracyclines daunoribicin (DNR) and doxorubicine (DOXO), anthracenedione mitoxantrone (MTX), and distinct chemotherapeutics cisplatin (CisPt) and paclitaxel (TAX) were used for evaluation. In the first dimension, untreated controls, CisPt and TAX were separated from DOXO, DNR and MTX treatments, the second and third dimensions evidently distinguished DNR from DOXO and MTX as well as CisPt and TAX.

### 2.7. Verification of Selected Protein Changes Using Immunoblot

**Figure 7:**
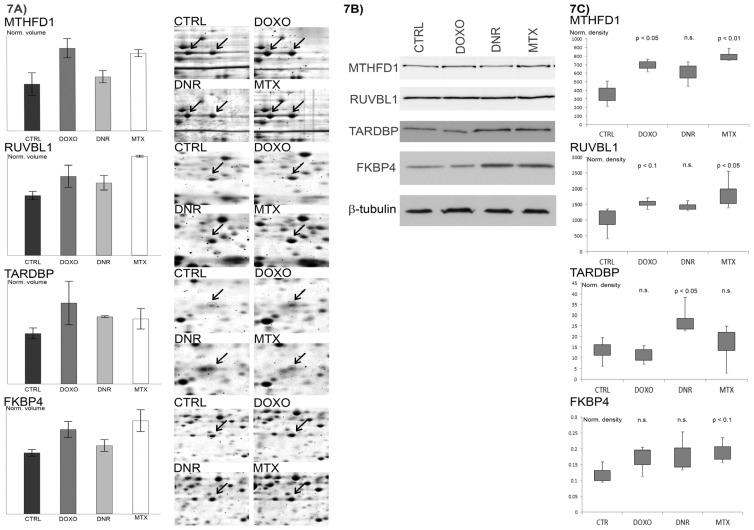
Western blot analysis of selected proteins.

Several of the differentially abundant proteins were selected for verification of 2DE observations using Western blot analysis (Figure 7). Among them, significant increase of MTHFD1 after both DOXO and MTX treatments was confirmed. Further, FKBP4, another increased protein on 2DE after both DOXO and MTX treatment, was also tracked using specific antibody and significant increase reflecting a probability of 90% was confirmed for MTX whilst DOXO showed a slight increase. Using 2-DE, RUVBL1, one of the proteins typical for MTX response, was significantly increased not only after MTX treatment but a non-significant increase observed after DOXO was shown as a significant change by immunoblot. Amongst the proteins typical for DNR response the transcription factor TARDBP was verified as significantly increased after DNR treatment.

Immunoblot analysis of *C*-1-tetrahydrofolate synthase (MTHFD1), RuvB-like 1 protein (RUVBL1), TAR DNA-binding protein 43 (TARDBP) and peptidyl-prolyl cis-*trans* isomerase FKBP4 (FKBP4) in untreated CEM cells and CEM cells treated with anthracycline drugs doxorubicin (DOXO), daunorubicin (DNR) and mitoxantrone (MTX) was performed. (7A) Bar plots of normalized volume intensities of selected differentially abundant protein spots calculated and graphically represented from 2D gels by Redfin software and corresponding protein features on 2D gels of control cells and drug treatments. Significantly regulated protein spots between control cells and individual treatments are in graphs indicated by an asterisk. The arrows indicate the location of each protein feature. (7B) The whole cell lysates were examined on immunoblot using specific antibodies directed against selected proteins. β-tubulin was used as a loading control. (7C) The protein bands from immunoblot were quantified using Quantity one software for at least three replicates analyzed per protein and the density of individual band was normalized for total density in given protein line. The results were illustrated as boxplots. Significance of differences between controls and each treatment was calculated using Student’s *t*-test for *p* < 0.05 and *p* < 0.01 (n.s., not significant).

## 3. Discussion

To gain insight into molecular mechanisms and biological processes underlying the treatments with representative anti-cancer anthracycline/anthracenedione drugs DNR, DOXO and MTX, we have used CEM T-lymphoblastic leukemia cells and investigated protein fingerprints of the drug effects employing combination of zoomed 2DE with fluorescent protein stain and MALDI-TOF/TOF mass spectrometry. The CEM T-lymphoblastic leukemia cells have been considered as suitable model of hematological malignancies as well as tumor cells sensitive to various anti-cancer drugs [18]. Several previous studies focused on the effects of DOXO or DNR with mostly applied 24 h or 48 h treatments and low micromolar concentrations of drugs, which may correspond to relevant clinical doses [11–15]. In our study, we designed proteomic experiments focused on earlier time intervals in order to reliably monitor protein alterations that precede induction of apoptosis and minimize its impact on observed protein changes. Using individual half time to onset of apoptosis (TA_50_), corresponding 10 times IC_50_ doses of the drugs instead of the same time interval for all treatments allowed us to optimize comparable stage of all used anti-cancer treatments. Whilst for four out of five drugs TA_50_ ranged from 120 min to 150 min, the longest 250 min interval was confirmed for DOXO and even this was still at least 6 times shorter than what was used in previously published studies [11–15].

To date, the effect of DOXO treatment on different cancer cell lines has mainly been studied by proteomic techniques [11,13,14]. To extend current observations and with the view to help translation of molecular findings toward improvements in clinical use, we focused on the effects of several clinically relevant representatives of the group of anthracycline/anthracenedione drugs. Hence, comprehensive proteome map of model T-lymphoblastic leukemia cells and its alterations after DNR, DOXO and MTX drug treatments were monitored and evaluated either by pair comparison to relevant untreated control or multivariate classification of drug treated and untreated samples.

In order to emphasise proteins specific for response toward anthracycline/anthracenedione drugs among all identified differentially abundant proteins, we performed in the same design, analysis of the effects of two additional anti-cancer drugs, CisPt and TAX, taken from distinct groups of chemotherapeutics, and compared protein alterations to those found after DNR, DOXO and MTX. As expected, using this step we marked the proteins affected and shared in anti-cancer response of such drug treatments. These proteins belong to the enzymes critical for cellular metabolism such as G6PD, the enzyme producing pentose sugars essential for nucleic acid synthesis; PHGDH, the enzyme involved in syntheses of purines and amino acids; NDUFS1, core subunit of the mitochondrial membrane respiratory chain NADH dehydrogenase (Complex I). More interestingly and corroborating our findings are the observations that many of these “promiscuous” general anti-cancer response proteins are the ones already known to play a vital role in various human cancers. For example PPME1 that demethylates protein phosphatase 2A was recently described as tumor suppressor [19]. TRA2B or HNRNPA2B1 regulating repair of double strand breaks have elevated levels in various cancers [20] and changed in levels by anti-cancer treatments as shown here. HNRNPA2B1 has been even assigned as proto-oncogene [21]. Further evidence is presented by KHSRP regulating transcription and mRNA processing which was shown to support migration in liver cancer cells [22]. Additionally, involvement of multifunctional protein PPIA in cancer progression has been described [23]. Interestingly, several cytoskeleton regulating proteins including CFL1 [24] and EZR [25] were associated with invasion and metastasis and ARHGDIB was linked to the development of chemoresistance [26]. These proteins, although non-specific as regards used drugs and functioning in various biological processes, most probably present important targets underlying anti-cancer mechanisms and possibly play role of anchor molecules which may connect different pathways in a very complex regulation of cancer cell processes. Despite their importance, the major aim of this study has been to identify specific proteins typical for the response to anthracycline/anthracenedione drugs DNR, DOXO and MTX and to characterize similarities in the effects of these structurally very close drugs.

In total, we found several tens of proteins with significantly changed levels at early time intervals after DNR, DOXO and MTX treatments which corresponded only to 1%–2% of the total number of spots detected. According to Gene Ontology classification of biological processes the highest representation of identified proteins for all three drugs belongs to metabolic processes of nucleic acids or proteins and cellular processes involved mainly in cytoskeleton organisation. It corresponds to well-known observations that metabolic alterations on glucose consumption and biosynthetic activity of nucleotides, amino acids and lipids are the changes for sustaining cell proliferation in cancer cells. Typical evidence of this fact is the Warburg effect, the conditions when the cancer cells switch from oxidative phosphorylation to glycolysis to produce ATP and set of enzymes such as lactate dehydrogenase and pyruvate dehydrogenase play crucial role [27]. Evidently and surprisingly, we observed in our study such changes in CEM T-lymphoblastic leukemia cells at very early time intervals after anti-cancer DNR treatment. The most probable explanation of this behaviour is adaptive effort of tumor cells to make even stronger the essential mechanisms supporting cancer growth. Regulation of metabolic enzymes offers new directions for anti-cancer treatments and lactate dehydrogenase which catalyses the final step in the glycolytic cascade constitutes a relatively new anti-cancer target [28]. Nevertheless, design of the combination of the enzymes or even their isoforms and development of specific inhibitors that would eliminate robustness of cancer cells is not a simple task.

In addition to changes in energy metabolism, DNR treatment of CEM cells leads to the decrease of two heterogeneous nuclear ribonucleoproteins which are involved in RNA processing but we also observed increase of TARDBP which is homologous to the heterogeneous nuclear ribonucleoproteins. The higher level of this protein was further confirmed using Western blot. The TARDBP has been identified as a cause of neuropathology in a wide spectrum of neurodegenerative diseases, including amyotrophic lateral sclerosis. Using Drosophila model for proteinopathy associated with TARDBP, it was shown that increasing human wild-type TARDBP expression is sufficient to cause neurotoxicity *in vivo*[29]. The protein may also be involved in microRNA biogenesis, apoptosis and cell division [30]. The finding of increased level of TARDBP in CEM leukemic cells after anti-cancer DNR treatment let us hypothesise that it might significantly contribute to the toxicity toward the tumor cell and positively influence outcome of anti-cancer response. Higher levels of this protein may also result from its decreased clearance, which was shown as mediated by lower activity of ubiqutin-proteasome system and autophagosome in synergy [31]. Hence, the link between the level of TARDBP and activity of ubiqutin-proteasome system and autophagosome is another good example underlying importance of these cellular mechanisms in regulation of carcinogenesis or response of cancer cell to anti-cancer treatment.

Doxorubicin, the other member of anthracyclines, also affected metabolic and cellular biological processes in CEM leukemic cells and majority of targeted proteins were exclusively specific for this drug and increased in cells after drug treatment. Among them, the role of spermidine synthase is aimed to redox regulation of tumor cell followed by anti-cancer treatment. Overproduction of spermidine increases resistance to oxidative stress with spermidine serving as a free-radical scavenger *in vitro* as well as *in vivo*[32]. Hence, increase of spermidine synthase in DOXO treated cancer cell may present regulatory response which may increase resistance of cancer cell.

EEF2 translates growth and stress impulses to the regulation of protein synthesis by catalyzing ribosomal translocation step during translation elongation. However, phosphorylation of EEF2 by EEF2 kinase inactivates this factor which indicates that EEF2 kinase could be promising anti-cancer target. Interestingly, using pharmacological inhibition of EEF2 kinase demonstrated that anti-cancer activity of widely accepted inhibitor and anti-proliferation agent against different cancer cells was more correlated with induction of EEF2 phosphorylation than inhibition of EEF2 kinase activity. In addition, stronger induction of EEF2 phosphorylation mediated by AMPK activators and mTOR inhibitor was linked to more effective cancer cell growth inhibition. Accordingly, EEF2 phosphorylation appears to be mediated through multiple pathways thus alarming the need of combinatory inhibition of EEF2 kinase in anti-cancer therapy [33]. In our study, we identified EEF2 in two protein spots from 2DE (Figure 4J). The more basic and less abundant spot was increased after DOXO treatment and may represent non-phosphorylated form, whilst the more acidic and more abundant protein spot may be representative of phosphorylated form increased by DOXO and MTX. The presence of more abundant/phosphorylated form might contribute to anti-cancer effect of DOXO and MTX, whilst the less abundant basic/non-phosphorylated form would have a role in regulation of protein synthesis and sustaining cancer cell growth.

One of a few examples of proteins decreased in level after DOXO treatment was found to be metabolic protein KHDRBS1. In case of human breast tumors it was shown that phosphorylation of this protein regulated its intracellular localization and anti-proliferative properties were blocked by phosphorylation [34]. Therefore, in addition to quantitative changes observed in this study, it would be necessary to investigate its post-translationally modified forms and localization as regards contribution to anti-cancer effect of DOXO.

Among the proteins of cellular processes affected by DOXO, we observed increase of UBA1 controlling ubiquitin conjugation pathway, and MCM7 having a role in DNA strand elongation involved in DNA replication. MCM7 is a known component of minichromosome maintenance complex which is the putative replicative helicase in eukaryotic cells and demonstrated to be efficient and sensitive marker to assess disease progression in the uterine cervix [35], prognosis of patients with non-small cell lung cancer [36], or Hodgkin lymphoma [37]. Comprehensive comparative analysis of pre-replication complex proteins in transformed and normal cells indicated that cellular transformation was associated with an overexpression and increased chromatin association of the pre-replication complex proteins including MCM7 [38]. From this point of view, increased level of MCM7 at early time interval after anti-cancer DOXO treatment may reflect other adaptive mechanisms of cancer cell contributing to the transformation of cell.

Transport proteins appeared to be an important group of proteins responding to DOXO treatment. They included SAR1B involved in protein transport from endoplasmic reticulum to Golgi, and cytoplasmic activation/proliferation-associated protein-1, CAPRIN1, stress granule associated protein. These findings may suggest possible role of induction of endoplasmic reticulum stress associated with proteotoxic stress. Subsequently, such stress stimulates either apoptosis of cancer cell which is involved in anti-cancer effects or autophagy as a cytoprotective, stress-induced adaptive pathway following disruption of protein homeostasis [39]. CAPRIN1 may also regulate the transport and translation of mRNAs of proteins with impact on cell proliferation and negative regulation of translation. The protein is putative target of miR-16 thus linking miRNA to the regulation of cell proliferation [40]. Overexpression of CAPRIN1 induced phosphorylation of eukaryotic translation initiation factor 2 alpha resulted in global inhibition of protein synthesis [41]. This may be synergistic with above mentioned role of phosphorylated EEF2 in suppression of protein synthesis as a part of anti-cancer effect of DOXO.

The majority of MTX induced protein alterations were moderate metabolic changes. Interesting, EIF2S3L which functions in the early steps of protein synthesis, PSMA5 and RUVBL1 with the roles in transcriptional regulation, DNA replication and probably DNA repair, were observed. Evidently, decrease of protein level mediated by decrease of EIF2S3L may play an important role in MTX anti-cancer effect. Furthermore, protein homeostasis which is controlled by ubiquitin–proteasome system as mentioned above seemed to be critical mechanisms for cancer cell. Pharmacologic inhibitors of the proteasome promote tumor cytotoxicity and clinical studies have showed improvement in patient survival. Despite success of the proteasome inhibitor bortezomib in the treatment of the hematologic malignancy such as multiple myeloma, treatment of the more complex solid tumors has been less successful [42]. Our results document that MTX similarly to DOXO exploit proteins of ubiquitin-proteasome system to trigger or modulate cancer cell stress response to anti-cancer treatment in order to induce either apoptosis or autophagy.

RUVBL1 is a highly conserved AAA(+) ATPase whose expression as well as expression of its homolog RUVBL2 was high in different cancers. In case of human hepatocellular carcinoma silencing of RUVBL2 reduced cell growth and increased apoptosis whilst overexpression enhances tumorigenicity [43]. The level of RUVBL1 was significantly increased in CEM cells treated with MTX, and Western blot analysis confirmed a significantly increased level not only after MTX but also DOXO drug treatment. The question remains whether RUVBL1 at an increased level is involved in promotion of tumorigenicity in CEM T-lymphoblastic cells similarly as described in the study on human hepatocellular carcinoma.

It was possible to see that very selective group of MTX treatment are the proteins of immune system process and response to stimuli, namely chaperones thus indicating significant contribution of protein folding and stress response in tumorigenesis and anti-cancer treatment [44]. Furthermore, these chaperone proteins may be involved in presentation of tumor antigens for direct recognition of tumor by T cells [45,46] or as autoantigens which can give raise to the production of autoantibodies [47]. As regards anthracyclines, Fucikova *et al.*[48] investigated the effectiveness of anthracyclines to induce immunogenic cell death in human tumor cell lines and primary tumor cells. The data demonstrated induction of immunogenic cell death in sensitive human tumor cells including human prostate cancer, ovarian cancer, and acute lymphoblastic leukemia cells treated by anthracyclines as anti-cancer drugs. Our findings of increased chaperone proteins after MTX treatment corroborate such published data and support the role of chaperons in tumor immunity.

This study has shown that each of the studied anti-cancer anthracycline/anthracenedione drugs possess typical proteins or protein variants which are specifically changed in level by individual drugs despite of their very close structural similarity which is currently used for their grouping within chemotherapeutic drugs. However, the design of our study allowed us to evaluate and classify proteome maps of all tested anti-cancer drugs to characterize the similarities that would link drug responses. Importantly, the observation of significant decrease of LDHB after treatment of anthracyclines DNR and DOXO as well as anthracenedione MTX thus underlies common anti-cancer effect of this group of drugs directed to the energy metabolism of cancer cell. Nevertheless, it has been important to be aware of the fact, as shown in several examples mentioned above, that the given drug may affect preferentially certain isoform/species of an individual protein hence, in many cases the specific role of such protein isoform/species may play decisive role compared to the quantitative change at the total level of a given protein.

Furthermore, we found several proteins common in DOXO and MTX, among them mainly those directed to the regulation protein synthesis as well as purine and amino acid biosynthesis including MTHFD1 whose increase after treatment by DOXO and MTX was confirmed by Western blot. Regulation of SFRS3 appeared to be a new emerging role because it was recently described as a proto-oncogene critical for cell proliferation and tumor induction and maintenance. It was highly expressed in various cancers and its reduction, mediated by RNAi, resulted in G2/M arrest, growth retardation, and apoptosis [49]. Accordingly, decreased level of SFRS3after DOXO and MTX treatments offers a new mechanism contributing to anti-cancer activities common to anthracycline/anthracenedione drugs.

Compared to a group of proteins linking the effect of DOXO and MTX, there were only a few proteins shared between DNR and DOXO or MTX thus indicating the distinct position of DNR among the anthracycline/antracenedione drugs. This finding was further corroborated by principal component analysis showing DNR sequestered from DOXO and MTX as well as other treatments such as CisPt and TAX in the first three components covering in total 71% of variances of the whole experimental set. Interestingly, there were two proteins, PSPC1 and HNRNPH3 which were shared between DNR/MTX and DNR/DOXO treatments, respectively, with surprisingly high fold changes observed. PSPC1 is required for the formation of nuclear paraspeckles, subnuclear bodies that alter gene expression via the nuclear retention of RNAs [50]. It belongs to the family of proteins of the Drosophila behavior/human splicing (DBHS) which are predominately nuclear and influence various biological processes, including carcinogenesis. The significant increase of PSPC1 after DNR and MTX treatments points to possible important role of nuclear paraspeckles in anti-cancer activities of anthracycline/anthracenedione drugs.

## 4. Materials and Methods

### 4.1. Cell Cultures and Sample Preparation—Determination of IC_50_ and TA_50_

Human T-lymphoblastic leukemia CEM cells (American Tissue Culture Collection, Manassas, VA, USA) were cultured at a density of 1 × 10^6^ cells/mL in RPMI-1640 medium supplemented with 2 mM glutamine, 100 U/mL penicillin, 100 μg/mL streptomycin, and 10% of heat inactivated fetal bovine serum with or without addition of anti-cancer drug in a humidified incubator with 5% CO_2_ at 37 °C. Drugs were dissolved directly in RPMI-1640 medium.

The cytotoxicity of DNR, DOXO, MTX, cisplatin (CisPt) and paclitaxel (TAX) was determined by the three-day MTT test as described previously and the inhibitory concentration corresponding to 50% of cell growth (IC_50_) was calculated [51]. Early time interval studies, when the influence of apoptosis is minimal, facilitate reliable observation of protein changes and hence time to apoptosis induction (TA) was measured for five times IC_50_ and 10 times IC_50_ doses of the drugs using caspase 3 and/or 7 activation Magic Red™ caspase detection kit [52]. For these relatively high drug doses used, no significant differences in TA for individual drugs were found. Hence, for proteomic analysis, the cells were treated with ten times IC_50_ doses of the drugs and harvested at half time to apoptosis induction (TA_50_) (Table 1). Cells were washed three times in ice-cold PBS and 6 × 10^6^ cells were lysed in 200 μL of lysis buffer containing 7 M urea, 2 M thiourea, 3% *w*/*v* CHAPS, 2% *v*/*v* Nonidet-P40, 5 mM TCEP in presence of inhibitors of proteases and phosphatases (Roche, Basel, Switzerland) according to manufacturers’ directions. After centrifugation at 4 °C, 20,000× *g*, 10 min, the supernatant was collected and protein concentration was determined by the Pierce 660 nm protein assay. Samples were frozen to −80 °C for future use. At least three biological replicates were analyzed for each drug treatment.

### 4.2. Two-Dimensional Gel Electrophoresis (2DE)

Aliquots of samples corresponding to 100 μg of proteins and 0.5% IPG buffer 4–7 were loaded on pH 4–7 Immobiline Drystrips using active in gel rehydration (IPG strip 18 cm, pH 4–7) in buffer containing 7 M urea, 2 M thiourea, 4% CHAPS, 200 mM DeStreak, inhibitors of proteases, phosphatases (Roche, Basel, Switzerland), 0.5% IPG buffer 4–7 and a trace of bromophenol blue. Isoelectric focusing separation (IEF) was performed on IEF Cell (Bio-Rad, Hercules, CA, USA) system using the following program: 1 h to 200 V, 10 h 200 V, 30 min to 500 V, 30 min to 1000 V, 1.5 h to 5000 V, and 5000 V until total of 55 kVh was reached. After IEF separation, the gel strips were equilibrated in 50 mM Tris, pH 6.8, 6 M urea, 30% glycerol, 4% SDS, 100 mM DeStreak, and a trace of bromophenol blue for 25 min [53].

Aliquots of samples corresponding to 70 μg of proteins and 0.5% IPG buffer 6–11 were cup-loaded on pH 6–11 Immobiline DryStrips (IPG strip 18 cm, pH 6–11) passively rehydrated in buffer containing 7 M urea, 2 M thiourea, 4% CHAPS, 30 mM DTT, inhibitors of proteases, phosphatases (Roche, Basel, Switzerland), 0.5% IPG buffer 6–11 and a trace of bromophenol blue overnight. IEF was performed on IEF Cell system using the following program: 1 h to 150 V, 12 h 150 V, 1 h to 1000 V, 3 h to 8000 V, and 8000 V for 12 kVh. After IEF separation, the strips were equilibrated in 50 mM Tris, pH 6.8, 6 M urea, 30% glycerol, 8% SDS, and 1% DTT for 15 min, followed by equilibration in 50 mM Tris, pH 6.8, 6 M urea, 30% glycerol, 8% SDS, 4% IAA and a trace of bromophenol blue for 15 min.

After equilibration, both 4–7 and 6–11 IPG strips were rinsed and applied to vertical 12% SDS-PAGE (18 mm × 18 mm × 1 mm gel). SDS-PAGE was carried out at a constant current of 40 mA per gel using in series connected Protean II xi Cells (Bio-Rad, Hercules, CA, USA) allowing simultaneous run of six gels. Gels were then stained with Sypro Ruby according to manufacturers’ directions. Stained gels were scanned and digitized at 50 μm resolution at Pharos FX fluorescent scanner (Bio-Rad, Hercules, CA, USA) with excitation length 488 nm and emission length 605 nm.

The images were evaluated using Redfin 3.3.2 Solo software (Ludesi, Malmo, Sweden, 2010). Pair comparison between untreated controls and each single treatment was performed for all 5 drugs. At least three biological replicates were used for each treatment or control samples. Protein spots that were statistically significant according to Student’s *t-*tests with *p* < 0.01 and fold-change >1.2 as well as *p* < 0.05 and fold-change >1.5 and accepted by visual inspection were selected for identification by mass spectrometry.

### 4.3. Protein Identification by Mass Spectrometry

Preparative 2D gels were prepared for spot excision and in gel digestion of selected protein spots according to the protocol above with the following modifications. Protein load was 500 μg of total protein amount per pH 4–7 gels and 150 μg of total protein amount per pH 6–11 gels. Gels were stained with reverse zinc staining [54]. Protein spots were excised from gels, cut into small pieces and destained using chelating agent. After complete destaining, the gel was washed with water, shrunk by dehydration in ACN and re-swollen again in water. The supernatant was removed and the gel was partly dried in a SpeedVac concentrator. The gel pieces were then rehydrated in a cleavage buffer containing 25 mM 4-ethylmorpholine acetate, 5% ACN and trypsin (5 ng/μL), and incubated overnight at 37 °C. The digestion was stopped by addition of 5% TFA in ACN and the aliquot of the resulting peptide mixture was desalted using a GELoader microcolumn (Eppendorf, Hamburg, Germany) packed with a Poros Oligo R3™ material [55]. The purified and concentrated peptides were eluted from the microcolumn in several droplets directly onto MALDI plate using 1 μL of *R*-cyano-4-hydroxycinnamic acid (CCA) matrix solution (5 mg/mL in 50% ACN/0.1% TFA).

Mass spectra were measured on an Ultraflex III MALDI-TOF/TOF instrument (Bruker Daltonics, Bremen, Germany) as described before [56]. Briefly, peptide mass fingerprint spectra were acquired in the mass range 700–3500 Da and peak list in XML format created using FlexAnalysis 3.0 searched using MASCOT search engine against Swiss-Prot 2011_09 database subset of human proteins with the following search settings; peptide tolerance of 50 ppm, missed cleavage site value set to one, variable carbamidomethylation of cysteine, oxidation of methionine and protein *N*-terminal acetylation. Proteins with Mascot score over the threshold 56 calculated for the used settings were considered as identified. If the score was lower or only slightly higher than the threshold value, the identity of protein candidate was confirmed by MS/MS analysis.

### 4.4. Western Blot Analysis

Protein lysates prepared as described above were diluted in 2× SDS buffer (4% SDS, 50% glycerol, 140 mM mercaptoethanol, 125 mM Tris pH 6.8 and a trace of bromophenol blue) and separated by 10%, 12% or 15% SDS-PAGE. Separated proteins were transferred onto Immobilone-P membrane (Millipore, Bedford, MA, USA) using a semidry blotting system (Biometra, Gottingen, Germany). The membranes were blocked in 5% non-fat dry milk or in 5% BSA in Tris-buffered saline with 0.05% Tween 20 (TBST) pH 7.4 for 1 h and incubated overnight with the respective primary antibodies: anti-MTHFD1 (Sigma-Aldrich, St. Louis, MO, USA) (HPA000704, 1:20000 in 5% BSA); anti-RUVBL1 (Sigma-Aldrich, St. Louis, MO, USA) (HPA019947, 1:200,00 in 5% BSA); anti-TARDBP (Sigma-Aldrich, St. Louis, MO, USA) (HPA017284, 1:1000 in 5% milk); anti-FKBP4 (Sigma-Aldrich, St. Louis, MO, USA) (HPA006148, 1:200,00 in 5% BSA) and anti-β-tubulin (Sigma-Aldrich, T4026, 1:5000 in 5% milk). Peroxidase-conjugated secondary anti-mouse or anti-rabbit antibodies (Jackson Immunoresearch, Suffolk, UK) were diluted 1:100,00 in 5% non-fat dry milk in TBST and incubated for 1 h at RT. The ECL+ chemiluminescence (GE Healthcare, Uppsala, Sweden) detection system was used to detect the proteins. The exposed CL-XPosure films (Thermo Scientific, Rockford, IL, USA) were scanned by a calibrated densitometer GS-800 (Bio-Rad, Hercules, CA, USA). QuantityOne 4.6.5. (Bio-Rad, Hercules, CA, USA, 2008) software was used for analysis and quantification of Western blot results.

### 4.5. Data Analysis Applying Principal Component Analysis (PCA)

Statistical analyses were performed using freeware R 2.14.1. (www.r-project.org) (R Foundation, Vienna, Austria, 2011). PCA was used to determine grouping of the drugs on the basis of similarities/differences in protein patterns. For each drug, arithmetical means of ratios measured intensity/volume of the spot were taken as input data [57].

### 4.6. Protein Classification According to Gene Ontology

The PANTHER (Protein ANalysis THrough Evolutionary Relationships) classification software was used to assign identified proteins according to Gene Ontology to biological processes (PANTHER version 7) [58].

## 5. Conclusions

To help translation of molecular findings toward improvements in clinical use we focused on the effects of several clinically relevant representatives of the group of anthracycline/anthracenedione drugs on the tumor cell.

It was evident that each of the drugs of anthracycline/anthracenedione group of chemotherapeutics was capable of inducing exclusively specific protein changes in tumor cells, many of which represent possible new molecular mechanisms contributing to anti-cancer activity. On the other hand, we observed several protein changes that corresponded to the adaptive effort of cancer cells to sustain growth. The findings of drug specific protein changes induced by structurally related drugs might help in explaining their different clinical use. Additionally, protein changes common in the drugs may be exploited for further enhancement of anti-cancer activities.

In summary, together with induction of anti-cancer activities, there may be significant benefits in blocking the activation of adaptive pathways in order to improve the outcomes of a specific treatment in cancer patients undergoing therapy.

## Supplementary Information



## Figures and Tables

**Table 1:** The list of studied anti-cancer drugs with their abbreviations, 10 times of inhibitory concentrations corresponding to 50% of cell growth (10× IC_50_ ) and half times to apoptosis induction (TA_50_) for 10× IC_50_ doses of individual drugs.

Anti-cancer drug	Abbreviation	Mechanism of action	10× IC_50_ (μg/mL)	TA_50_ (min)
Daunorubicin	DNR	intercalation, topo II inhibitor	0.03	120
Doxorubicin	DOXO	intercalation, topo II inhibitor	0.05	250
Mitoxantrone	MTX	intercalation, topo II inhibitor	1.88 × 10^−3^	150
Cisplatin	CisPt	alkylating-like	7.57	150
Paclitaxel	TAX	mitotic inhibitor	9.00 × 10^−5^	120

**Table 2:** The list of studied anti-cancer drugs with the numbers of significant protein spot changes, direction of their changes and the number of identified proteins. DNR, daunorubicin; DOXO, doxorubicin; MTX, mitoxantrone; CisPt, cisplatin; TAX, paclitaxel.

Anti-cancer drug	No. Different Spot	4–7		No. ID proteins	No. Different Spot	6–11		No. ID proteins	No. Different Spot	Total		No. ID proteins
		
		Up Down				Up Down				Up Down		
DNR	32	8	24	24	8	1	7	5	40	9	31	29
DOXO	29	15	14	21	18	13	5	10	47	28	19	31
MTX	40	24	16	30	14	9	5	10	54	33	21	40
CisPt	24	21	3	18	23	16	7	13	47	37	10	31
TAX	29	25	4	21	2	1	1	1	31	26	5	22
Total	154	93	61	114	65	40	25	39	219	133	86	153

**Table 3:** The list of identified changed proteins.

3A	DNR								

Drug	Spot No.	Protein name	Gene Name	UniProt No.	Biological process	pH	Change	Fold change	*p*-value
DNR	4	l-lactate dehydrogenase B chain	*LDHB*	P07195	3	4–7	↑	1.34	0.0048
DNR	7	Rho GDP-dissociation inhibitor 2	*ARHGDIB*	P52566	1	4–7	↑	1.69	0.008
DNR	36	Stathmin	*STMN1*	P16949	1	4–7	↑	1.75	0.0022
DNR	64	60 kD a heat shock protein, mitochondrial	*HSPD1*	P10809	5	4–7	↓	1.52	0.0023
DNR	72b	Proteasome subunit beta type-2	*PSMB2*	P49721	5	6–11	↓	1.70	0033
DNR	96b	Obg-like ATPase 1	*OLA1*	Q9NTK5	8	6–11	↑	1.34	0.0022
DNR	97	Cytochrome b-c1 complex subunit 1, mitochondrial	*UQCRC1*	P31930	3	4–7	↓	1.36	0.0041
DNR	107b	Glucose-6-phosphate 1-dehydrogenase	*G6PD*	P11413	5	6–11	↓	2.95	0.0013
DNR	124b	*S*-formylglutathione hydrolase	*ESD*	P10768	8	6–11	↓	2.22	0.009
DNR	125	Heat shock 70 kDa protein 4	*HSPA4*	P34932	4	4–7	↓	2.25	0.0076
DNR	126	Cofilin-1	*CFL1*	P23528	1	4–7	↑	2.01	0.0045
DNR	159	Succinyl-CoA: 3-ketoacid-coenzyme A transferase 1, mitochondrial	*OXCT1*	P55809	5	4–7	↓	1.67	0.0059
DNR	166	Dihydrolipoyllysine-residue acetyltransferase component of pyruvate dehydrogenase complex, mitochondrial	*DLAT*	P10515	5	4–7	↓	2.34	0.0004
DNR	220	Heterogeneous nuclear ribonucleoprotein H	*HNRNPH1*	P31943	5	4–7	↓	1.56	0.0017
DNR	221	Plastin-2	*LCP1*	P13796	1	4–7	↓	1.63	0.0093
DNR	287	DnaJ homolog subfamily A member 2	*DNAJA2*	O60884	4	4–7	↓	1.63	0.0021
DNR	346	Transitional endoplasmic reticulum ATPase	*VCP*	P55072	7	4–7	↓	2.55	0.0098
DNR	360	Mitochondrial-processing peptidase subunit alpha	*PMPCA*	Q10713	3	4–7	↓	1.66	0.0029
DNR	399	Anamorsin	*CIAPIN1*	Q6FI81	8	4–7	↓	1.47	0.0049
DNR	407	Protein phosphatase methylesterase 1	*PPME1*	Q9Y570	5	4–7	↑	1.44	0.0083
DNR	424	ATP synthase subunit beta, mitochondrial	*ATP5B*	P06576	3	4–7	↓	2.21	0.0009
DNR	573	60 kDa heat shock protein, mitochondrial	*HSPD1*	P10809	5	4–7	↓	1.72	0.0009
DNR	574	TAR DNA-binding protein 43	*TARDBP*	Q13148	5	4–7	↑	1.33	0.0065
DNR	768	Glutathione synthetase	*GSS*	P48637	5	4–7	↓	2.00	0.0019
DNR	849	Heterogeneous nuclear ribonucleoprotein F	*HNRNPF*	P52597	5	4–7	↓	1.71	0.009

**3B**	**DOXO**								

DOXO	29	Heat shock 70 kDa protein 1A/1B	*HSPA1A/HSPA1B*	P08107	4	4–7	↑	1.43	0.0073
DOXO	44b	Far upstream element-binding protein 2	*KHSRP*	Q92945	5	6–11	↑	1.53	0.0079
DOXO	61b	KH domain-containing, RNA-binding, signal transduction-associated protein 1	*KHDRBS1*	Q07666	5	6–11	↓	1.78	0.0004
DOXO	63b	EH domain-containing protein 1	*EHD1*	Q9H4M9	7	6–11	↑	1.79	0.049
DOXO	91	DNA replication licensing factor MCM7	*MCM7*	P33993	1	4–7	↑	1.56	0.0081
DOXO	115b	Elongation factor 2	*EEF2*	P13639	5	6–11	↑	1.54	0.0023
DOXO	141	Caprin-1	*CAPRIN1*	Q14444	7	4–7	↑	1.54	0.0008
DOXO	163b	Medium-chain specific acyl-CoA dehydrogenase, mitochondrial	*ACADM*	P11310	3	6–11	↓	1.45	0.0025
DOXO	170b	Far upstream element-binding protein 2	*KHSRP*	Q92945	5	6–11	↑	1.89	0.0013
DOXO	278	Spermidine synthase	*SRM*	P19623	5	4–7	↑	1.64	0.0049
DOXO	282	Ubiquitin-like modifier-activating enzyme 1	*UBA1*	P22314	1	4–7	↑	1.97	0.0062
DOXO	308	F-actin-capping protein subunit alpha-2	*CAPZA2*	P47755	1	4–7	↑	1.45	0.0098
DOXO	364	4-trimethylaminobutyraldehyde dehydrogenase	*ALDH9A1*	P49189	5	4–7	↑	1.51	0.0033
DOXO	592	Ezrin	*EZR*	P15311	1	4–7	↑	2.59	0.0071
DOXO	595	l-aminoadipate-semialdehyde dehydrogenase-phosphopantetheinyl transferase	*AASDHPPT*	Q9NRN7	8	4–7	↓	1.54	0.0009
DOXO	630	Ubiquitin-like domain-containing CTD phosphatase 1	*UBLCP1*	Q8WVY7	8	4–7	↓	1.36	0.0025
DOXO	699	Rho GDP-dissociation inhibitor 2	*ARHGDIB*	P52566	1	4–7	↓	2.00	0.0015
DOXO	787	Glutathione *S*-transferase P	*GSTP1*	P09211	4	4–7	↓	1.54	0.0089
DOXO	908	GTP-binding protein SAR1b	*SAR1B*	Q9Y6B6	7	4–7	↓	1.33	0.0073

**3C**	**MTX**								

MTX	11	Elongation factor 1-gamma	*EEF1G*	P26641	5	4–7	↑	1.32	0.0056
MTX	12	Protein disulfide-isomerase A3	*PDIA3*	P30101	5	4–7	↑	1.48	0.0056
MTX	22	Heterogeneous nuclear ribonucleoprotein F	*HNRNPF*	P52597	5	4–7	↑	1.37	0.0011
MTX	24	Stress-70 protein, mitochondrial	*HSPA9*	P38646	4	4–7	↑	1.47	0.007
MTX	25	60 S acidic ribosomal protein P0	*RPLP0*	P05388	5	4–7	↑	1.3	0.0012
MTX	30b	Staphylococcal nuclease domain-containing protein 1	*SND1*	Q7KZF4	5	6–11	↑	1.53	0.024
MTX	35	T-complex protein 1 subunit beta	*CCT2*	P78371	5	4–7	↑	1.47	0.0017
MTX	36b	Peptidyl-prolyl cis-*trans* isomerase	*PPIA*	P62937	4	6–11	↓	1.75	0.0072
MTX	68	T-complex protein 1 subunit gamma	*CCT3*	P49368	5	4–7	↑	1.56	0.00014
MTX	83	Activator of 90 kDa heat shock protein ATPase homolog 1	*AHSA1*	O95433	4	4–7	↑	1.47	0.00035
MTX	109b	Heterogeneous nuclear ribonucleoproteins A2/B1	*HNRNPA2B1*	P22626	5	6–11	↓	1.87	0.0071
MTX	110b	ATP-dependent RNA helicase DDX1	*DDX1*	Q92499	5	6–11	↑	1.51	0.008
MTX	116	RuvB-like 1	*RUVBL1*	Q9Y265	5	4–7	↑	1.66	0.00004
MTX	128b	Eukaryotic translation initiation factor 2 subunit 3	*EIF2S3L*	Q2VIR3	5	6–11	↓	1.73	0.02
MTX	131	60 kDa heat shock protein, mitochondrial	*HSPD1*	P10809	5	4–7	↑	1.52	0.001
MTX	136	Transaldolase	*TALDO1*	P37837	5	4–7	↑	1.33	0.0042
MTX	223	Mitochondrial inner membrane protein	*IMMT*	Q16891	8	4–7	↑	1.58	0.0028
MTX	230	Proteasome subunit alpha type-5	*PSMA5*	P28066	5	4–7	↑	2.51	0,004
MTX	297	Heat shock 70 kDa protein 1A/1B	*HSPA1A/HSPA1B*	P08107	4	4–7	↑	1.36	0.0066
MTX	455	G-rich sequence factor 1	*GRSF1*	Q12849	5	4–7	↑	1.66	0.0016
MTX	461	Rho GDP-dissociation inhibitor 2	*ARHGDIB*	P52566	1	4–7	↓	1.46	0.0033
MTX	466	Eukaryotic translation initiation factor 3 subunit G	*EIF3G*	O75821	5	4–7	↓	1.46	0.002
MTX	615	Transformer-2 protein homolog beta	*TRA2B*	P62995	5	4–7	↓	1.93	0.0025
MTX	647	Mitochondrial inner membrane protein	*IMMT*	Q16891	8	4–7	↑	1.34	0.0006
MTX	679	Stathmin	*STMN1*	P16949	1	4–7	↓	2.26	0.0028
MTX	1050	Adenylosuccinate synthetase isozyme 2	*ADSS*	P30520	5	4–7	↑	1.52	0.00005

**3D**	**DNR + DOXO**								

DNR	15b	Heterogeneous nuclear ribonucleoprotein H3	*HNRNPH3*	P31942	5	6–11	↓	1.88	0.04
DOXO	15b	Heterogeneous nuclear ribonucleoprotein H3	*HNRNPH3*	P31942	5	6–11	↓	2.25	0.025
DNR	279	Protein disulfide-isomerase A3	*PDIA3*	P30101	5	4–7	↓	1.50	0.0056
DOXO	279	Protein disulfide-isomerase A3	*PDIA3*	P30101	5	4–7	↓	1.59	0.004

**3E**	**DNR + MTX**								

DNR	320	Heat shock protein 75 kDa, mitochondrial	*TRAP1*	Q12931	4	4–7	↓	1.66	0.001
MTX	320	Heat shock protein 75 kDa, mitochondrial	*TRAP1*	Q12931	4	4–7	↑	1.33	0.0045
DNR	413	NADH-ubiquinone oxidoreductase 75 kDa subunit, mitochondrial	*NDUFS1*	P28331	3	4–7	↓	1.37	0.0015
MTX	413	NADH-ubiquinone oxidoreductase 75 kDa subunit, mitochondrial	*NDUFS1*	P28331	3	4–7	↑	1.6	0.0073
DNR	642	Paraspeckle component 1	*PSPC1*	Q8WXF1	5	4–7	↓	4.11	0.0028
MTX	642	Paraspeckle component 1	*PSPC1*	Q8WXF1	5	4–7	↓	2.44	0.0077

**3F**	**DOXO + MTX**								

DOXO	4b	Elongation factor 2	*EEF2*	P13639	5	6–11	↑	1.47	0.00038
MTX	4b	Elongation factor 2	*EEF2*	P13639	5	6–11	↑	1.47	0.00035
DOXO	25b	Peptidyl-prolyl cis-*trans* isomerase	*PPIA*	P62937	4	6–11	↓	1.85	0.0023
MTX	25b	Peptidyl-prolyl cis-*trans* isomerase	*PPIA*	P62937	4	6–11	↓	1.7	0.0025
DOXO	33b	*C*-1-tetrahydrofolate synthase, cytoplasmic	*MTHFD1*	P11586	5	6–11	↑	1.77	0.013
MTX	33b	*C*-1-tetrahydrofolate synthase, cytoplasmic	*MTHFD1*	P11586	5	6–11	↑	1.67	0.011
DOXO	37b	*C*-1-tetrahydrofolate synthase, cytoplasmic	*MTHFD1*	P11586	5	6–11	↑	1.69	0.0074
MTX	37b	*C*-1-tetrahydrofolate synthase, cytoplasmic	*MTHFD1*	P11586	5	6–11	↑	1.62	0.0122
DOXO	45	Peptidyl-prolyl cis-*trans* isomerase FKBP4	*FKBP4*	Q02790	4	4–7	↑	1.38	0.0066
MTX	45	Peptidyl-prolyl cis-*trans* isomerase FKBP4	*FKBP4*	Q02790	4	4–7	↑	1.53	0.0052
DOXO	46b	GMP synthase [glutamine-hydrolyzing]	*GMPS*	P49915	5	6–11	↑	1.6	0.014
MTX	46b	GMP synthase [glutamine-hydrolyzing]	*GMPS*	P49915	5	6–11	↑	1.65	0.0038
DOXO	70	Actin, cytoplasmic 1	*ACTB*	P60709	1	4–7	↓	1.38	0.0017
MTX	70	Actin, cytoplasmic 1	*ACTB*	P60709	1	4–7	↓	1.47	0.0009
DOXO	85b	DAZ-associated protein 1	*DAZAP1*	Q96EP5	6	6–11	↓	1.71	0.0011
MTX	85b	DAZ-associated protein 1	*DAZAP1*	Q96EP5	6	6–11	↓	2.22	0.0059
DOXO	140b	Far upstream element-binding protein 2	*KHSRP*	Q92945	5	6–11	↑	1.68	0.035
MTX	140b	Far upstream element-binding protein 2	*KHSRP*	Q92945	5	6–11	↑	1.61	0.0131
DOXO	153	d-3-phosphoglycerate dehydrogenase	*PHGDH*	O43175	5	4–7	↑	1.68	0.0068
MTX	153	d-3-phosphoglycerate dehydrogenase	*PHGDH*	O43175	5	4–7	↑	1.9	0.0097
DOXO	242	Splicing factor, arginine/serine-rich 3	*SFRS3*	P84103	5	4–7	↓	1.31	0.0064
MTX	242	Splicing factor, arginine/serine-rich 3	*SFRS3*	P84103	5	4–7	↓	1.58	0.0034
DOXO	526	Rab GDP dissociation inhibitor beta	*GDI2*	P50395	1	4–7	↓	2.08	0.0009
MTX	526	Rab GDP dissociation inhibitor beta	*GDI2*	P50395	1	4–7	↓	1.51	0.0052
DOXO	541	Heat shock protein 105 kDa	*HSPH1*	Q92598	4	4–7	↑	1.47	0.0028
MTX	541	Heat shock protein 105 kDa	*HSPH1*	Q92598	4	4–7	↑	1.75	0.0015

**3G**	**DNR + DOXO + MTX**								

DOXO	437	l-lactate dehydrogenase B chain	*LDHB*	P07195	3	4–7	↓	1.83	0.0064
MTX	437	l-lactate dehydrogenase B chain	*LDHB*	P07195	3	4–7	↓	1.86	0.0048
DNR	437	l-lactate dehydrogenase B chain	*LDHB*	P07195	3	4–7	↓	2.50	0.0032

Identified proteins typical for individual treatment with 3A: daunorubicin (DNR); 3B: doxorubicin (DOXO); 3C: mitoxantrone (MTX). Proteins with overlap between DNR and DOXO are listed in 3D; between DNR and MTX in 3E; between DOXO and MTX in 3F. Proteins with overlap between all three drugs are in 3G. Proteins from basic pH 6–11 are annotated as “b” beside spot number. The Gene Ontology biological process classification using PANTHER software is indicated by numbers 1 for cellular process, 2 for developmental process, 3 for generation of precursor metabolites and energy, 4 for immune system process/response to stimulus, 5 for metabolic process, 6 for reproduction, 7 for transport and 8 for un-annotated proteins.
