# Supplementary material for: Cancer Cell Response to Anthracyclines Effects: Mysteries of the Hidden Proteins Associated with These Drugs
Source: Int J Mol Sci. 2012 Nov 22;13(12):15536–64. doi: 10.3390/ijms131215536 (PMC3546648; doi:10.3390/ijms131215536)
Supplement: Supplementary file 1 [file ijms-13-15536-s001.pdf]

Supplementary Information

Figure S1. Graphical representation of identified differentially abundant proteins after doxorubicin (DOXO), daunorubicin (DNR), mitoxantrone (MTX), cisplatin (CisPt) and paclitaxel (TAX) treatment of CEM T-lymphoblastic leukemia cells. Light blue squares represent anti-cancer drugs. The nodes show identified proteins marked according to their gene names, the color code represents Gene Ontology biological based on PANTHER classification. The node shape shows trend of change in protein level, proteins with increased levels are depicted as triangles, proteins with decreased levels as arrowheads and proteins with opposite changes between different drugs as diamonds. Detailed information about the proteins is shown in Table 3 and Supplemental Table 2.

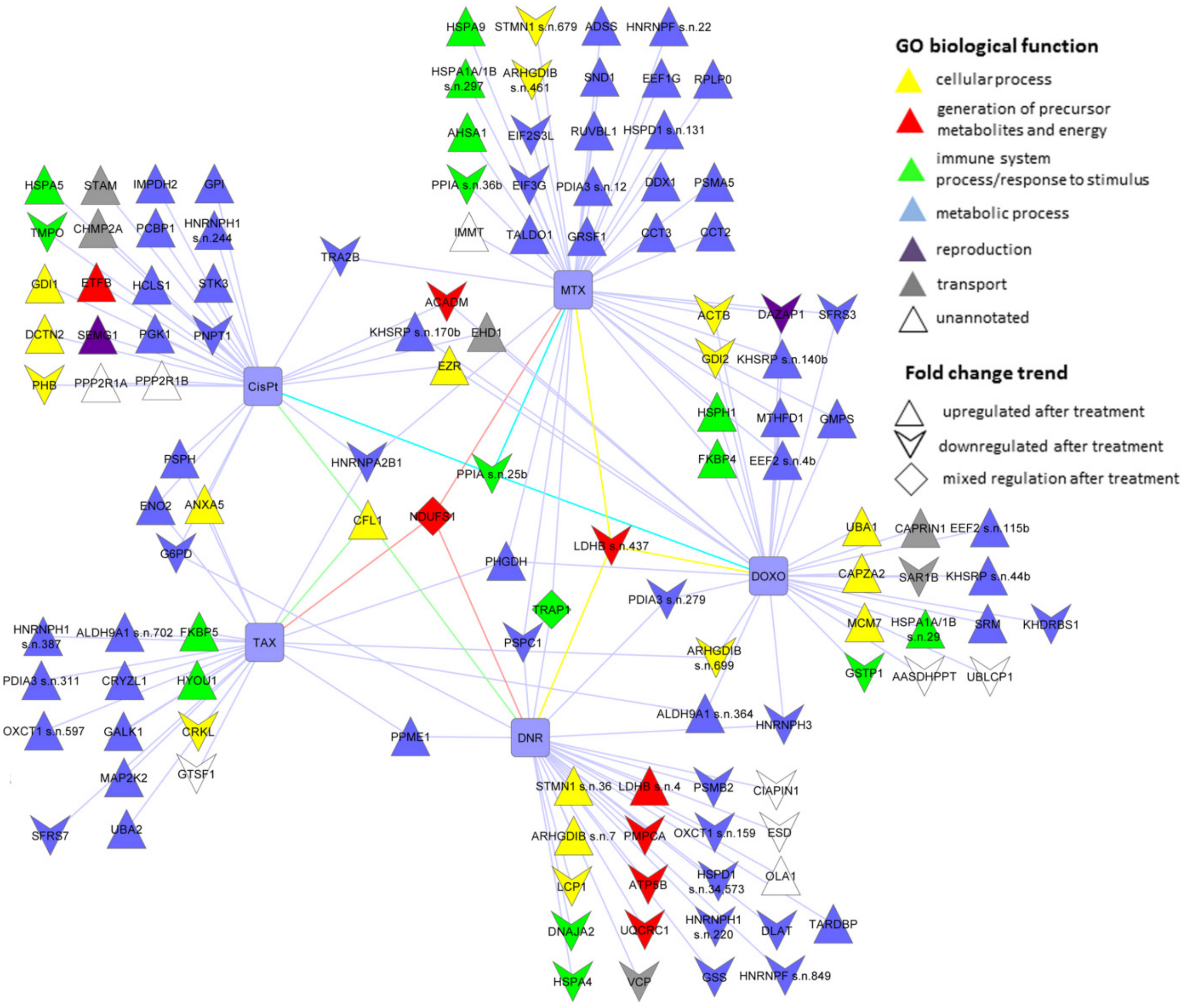

© 2012 by the authors; licensee MDPI, Basel, Switzerland. This article is an open access article distributed under the terms and conditions of the Creative Commons Attribution license (http://creativecommons.org/licenses/by/3.0/).
